# Supplementary figures and images for: Human RAD52 stimulates the RAD51-mediated homology search
Source: Life Sci Alliance. 2023 Dec 11;7(3):e202201751. doi: 10.26508/lsa.202201751 (PMC10713436; doi:10.26508/lsa.202201751)

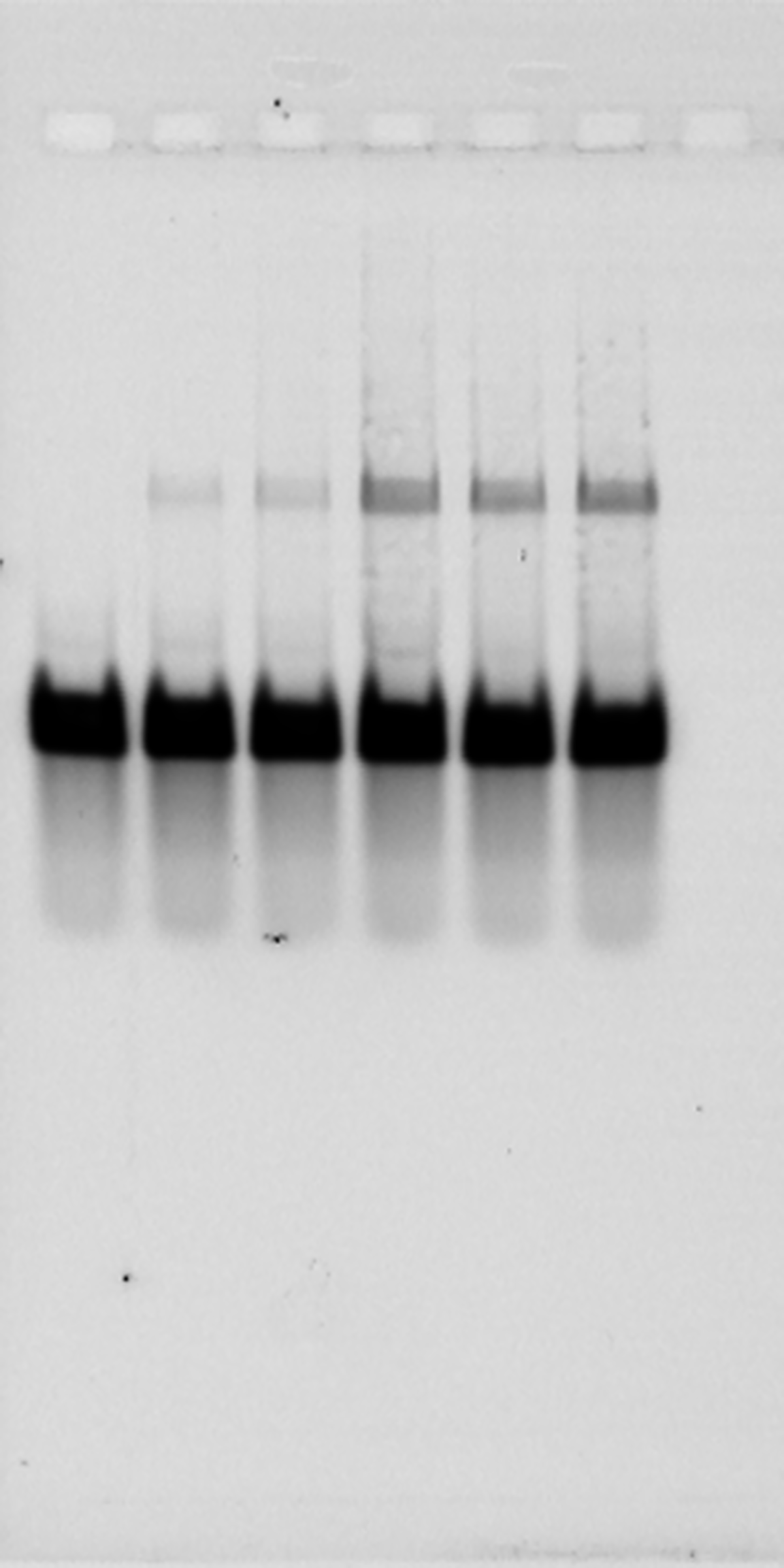

Supplement: Supplementary file 1 [file LSA-2022-01751_SdataF5.tif]
